# Poster Session II - A284 RISK OF COLECTOMY AFTER APPENDECTOMY IN ESTABLISHED ULCERATIVE COLITIS: A SYSTEMIC REVIEW AND META-ANALYSIS

**DOI:** 10.1093/jcag/gwaf042.284

**Published:** 2026-02-13

**Authors:** S Samnani, S Siyad, H AlAwadhi, N Narula

**Affiliations:** McMaster University, Hamilton, ON, Canada; McMaster University, Hamilton, ON, Canada; McMaster University, Hamilton, ON, Canada; McMaster University, Hamilton, ON, Canada

## Abstract

**Background:**

Ulcerative colitis (UC) is a chronic inflammatory bowel disease characterized by relapsing-remitting inflammation of the colon and rectum. Current management focuses on inducing and maintaining clinical and endoscopic remission to prevent complications like colectomy. Recent studies suggest the appendix may play an immunomodulatory role. Several studies have suggested those with appendectomy may be at lower risk of developing UC. Preliminary studies indicate there may even be a role for appendectomy in those with established UC, but its clinical effectiveness in established UC remains unclear.

**Aims:**

This systematic review and meta-analysis aims to evaluate the association of appendectomy and longer-term risk of colectomy in patients with established UC.

**Methods:**

We searched MEDLINE, EMBASE, and Cochrane Library from inception without date restrictions, including randomized controlled trials and observational studies reporting colectomy rates in patients with established UC who had appendectomy after their diagnosis of UC compared to patients without appendectomy. Primary outcome was rate of colectomy after appendectomy. Secondary outcomes included flare frequency, treatment response, need for steroids or change in medications. Data were synthesized using random-effects meta-analysis, with heterogeneity assessed via I2 and chi-squared tests. Study quality was evaluated using the Newcastle-Ottawa Scale and Cochrane Risk of Bias 2 tools.

**Results:**

We included 10 studies (1 randomized controlled trial (RCT) and 9 observational studies) (Table 1) from 1120 citations. Seven studies were eligible for inclusion with the meta-analysis, with significant heterogeneity (I2 = 78.02%, p = 0.0001). There was no significant association between appendectomy and odds of colectomy (OR 1.265, 95% CI 0.588–2.721, p = 0.547) (Table 2). Subgroup analyses by disease activity (active vs. remission) and UC extent were limited by data variability.

**Conclusions:**

Current evidence does not suggest a therapeutic benefit for appendectomy to lower rates of colectomy in those with established UC. Further data is needed to understand its role for induction and maintenance of remission.

A284 Table 1: Baseline characteristics of the included studies

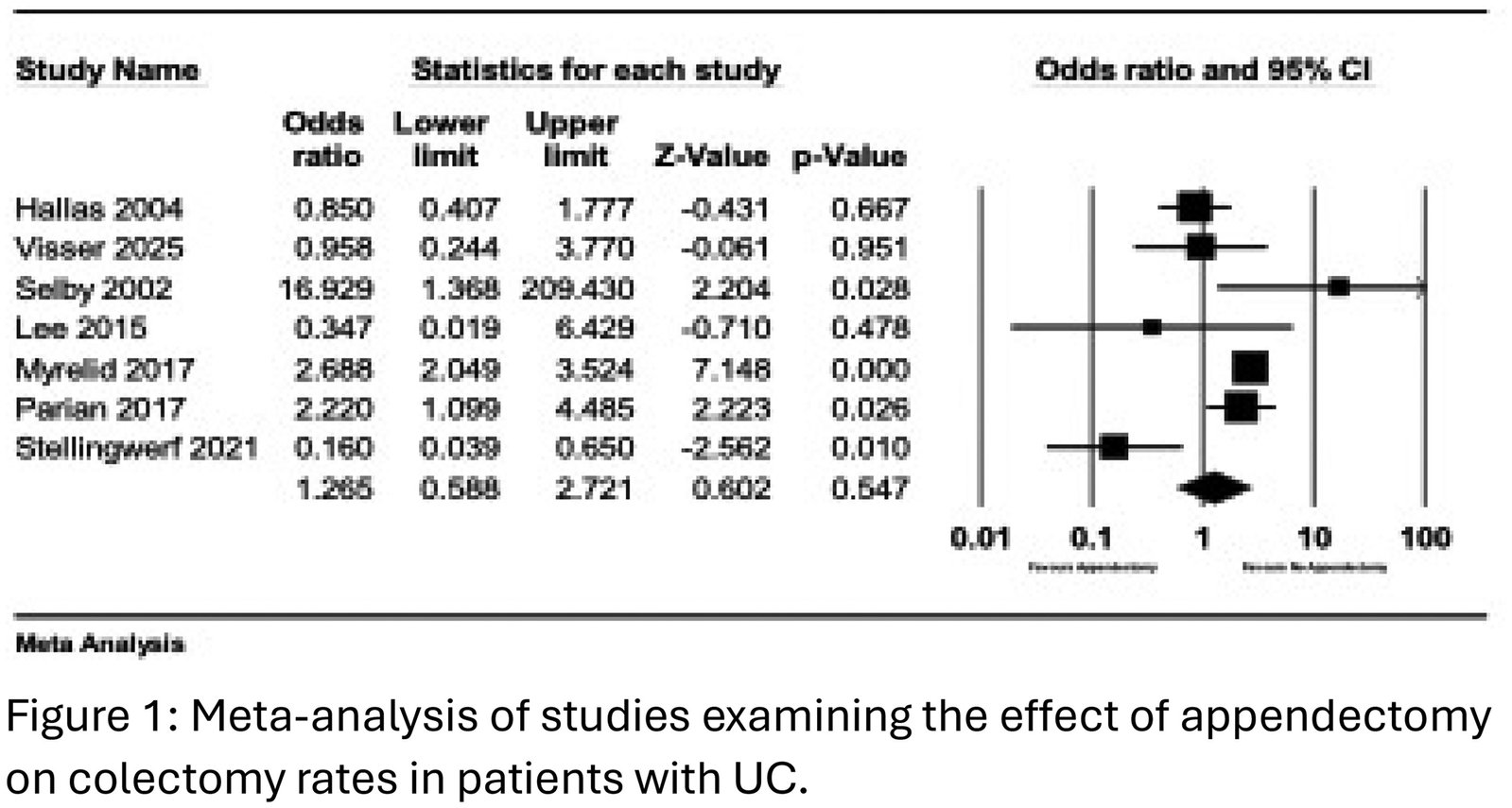

**Funding Agencies:**

None

**Table:** 

Author Last Name, Year	Location of Study	Study Design	Disease Status	Total number of participants	Mean age ± SD or median age [IQR] by group	Male n (%)
Acherman, 2025	Netherlands, Ireland, and the UK.	RCT	Remission	197 (99 appendectomy; 98 control)	Appendectomy: 33.7 ± 11.0; Control: 35.5 ± 12.6	Appendectomy: 43 (43%); Control: 43 (44%)
Hallas, 2004	Denmark	Reterospective	Remission	1,010 (202 appendectomy; 808 control)	Appendectomy: 38.6 ± 18.0; Control: 38.7 ± 17.7	Appendectomy: 84 (42%). Control: 336 (42%)
Visser, 2025	Netherlands	Prospective Cohort	Mod-severe UC	115 (65 appendectomy; 50 JAKi)	Appendectomy: 39 ± 14; Control: 43 ± 14	Appendectomy: 23 (35.4%); JAKi: 27 (54%)
Stellingwerf, 2019	Netherlands and Ireland	Prospective Cohort	Refractory UC	28 (appendectomy only; no control group)	40.5 [33.3–47.8]	15 (53.5%)
Selby, 2002	Australia	Prospective Cohort	Not reported	247 (8 appendectomy; 239 no appendectomy)	Appendectomy: 24.6 ± 3.44. No appendectomy: 32.1 ± 0.84	Not reported
Stellingwerf, 2021	Netherlands	Prospective Cohort	Not reported	33	No specific data for these 33 patients can be retrieved	No specific data for these 33 patients can be retrieved
Sahami, 2019	Netherlands and Ireland	Prospective Cohort	Refractory UC	30 (appendectomy cohort; no control group)	40 [33–47]	16 (53%)
Lee, 2015	Korea	Reterospective + nested Case-control	Not reported	180 (36 appendectomy; 144 matched controls)	Appendectomy 35 [11–73]; Controls 36 [11–80]	Appendectomy 19 (52.8%); Controls 75 (52.8%)
Myrelid, 2017	Sweden	National cohort; matched case–control analysis for post-diagnosis	Not reported	42,583 (603 appendectomy; 41,980 matched controls)	Appendectomy 33.6 ± 16.9 mean; Controls 42.4 ± 19.1	Appendectomy 300 (50%); Controls 22,148 (53%)
Parian, 2017	USA, Puerto Rico, Canada	Retrospective cohort with meta-analysis	Remission	2,651 (48 appendectomy; 2,603 no appendectomy)	Appendectomy 36.5 ± 15.9. No appendectomy 30.8 ± 14.5	Appendectomy 49 (44%). No appendectomy 1,320 (51%)

